# Linoleic Acid‐Rich Oil Alters Circulating Cardiolipin Species and Fatty Acid Composition in Adults: A Randomized Controlled Trial

**DOI:** 10.1002/mnfr.202101132

**Published:** 2022-06-21

**Authors:** Rachel M. Cole, Austin Angelotti, Genevieve C. Sparagna, Ai Ni, Martha A. Belury

**Affiliations:** ^1^ Program of Human Nutrition, The Department of Human Sciences The Ohio State University Columbus OH 43210 USA; ^2^ Division of Cardiology The Department of Medicine University of Colorado Anschutz Medical Center Aurora CO 80045 USA; ^3^ Division of Biostatistics College of Public Health The Ohio State University Columbus OH 43210 USA

**Keywords:** cardiolipin, cardiometabolic disease, dietary oils, fatty acid composition, linoleic acid, mitochondria, oleic acid

## Abstract

**Scope:**

Higher circulating linoleic acid (LA) and muscle‐derived tetralinoleoyl‐cardiolipin (LA_4_CL) are each associated with decreased cardiometabolic disease risk. Mitochondrial dysfunction occurs with low LA_4_CL. Whether LA‐rich oil fortification can increase LA_4_CL in humans is unknown. The aims of this study are to determine whether dietary fortification with LA‐rich oil for 2 weeks increases: 1) LA in plasma, erythrocytes, and peripheral blood mononuclear cells (PBMC); and 2) LA_4_CL in PBMC in adults.

**Methods and results:**

In this randomized controlled trial, adults are instructed to consume one cookie per day delivering 10 g grapeseed (LA‐cookie, *N* = 42) or high oleate (OA) safflower (OA‐cookie, *N* = 42) oil. In the LA‐cookie group, LA increases in plasma, erythrocyte, and PBMC by 6%, 7%, and 10% respectively. PBMC and erythrocyte OA increase by 7% and 4% in the OA‐cookie group but is unchanged in the plasma. PBMC LA_4_CL increases (5%) while LA_3_OA_1_ CL decreases (7%) in the LA‐cookie group but are unaltered in the OA‐cookie group.

**Conclusions:**

LA‐rich oil fortification increases while OA‐oil has no effect on LA_4_CL in adults. Because LA‐rich oil fortification reduces cardiometabolic disease risk and increases LA_4_CL, determining whether mitochondrial dysfunction is repaired through dietary fortification is warranted.

## Graphical abstract

This is the first randomized placebo‐controlled trial to show that fortifying the diet with a modest amount of linoleic acid rich oil increases linoleic acid in all three blood fractions, e.g., erythrocytes, plasma, and peripheral blood monocytes (PBMC). In addition, LA fortification increases PBMC levels of tetralinoleoyl‐cardiolipin, an inner mitochondrial membrane phospholipid that optimizes mitochondrial respiration.

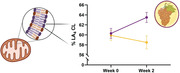

**Abbreviations**
LA: linoleic acid
MUFA: monounsaturated fatty acids
PBMC: Peripheral blood mononuclear cells
LA_4_ CL: tetralinoleoyl cardiolipin

## 1 Introduction

Dietary polyunsaturated fatty acid (PUFA) intake is associated with reduced cardiometabolic disease risk.^[^ ^1^, ^2^, ^3^, ^4^, ^5^, ^6^ ^]^ In addition, higher LA biomarkers have been associated with a reduced risk for cardiometabolic diseases and conditions, e.g., stroke,^[^ ^7^ ^]^ type 2 diabetes,^[^ ^8^, ^9^ ^]^ metabolic syndrome (MetS),^[^ ^10^ ^]^ visceral adipose,^[^ ^11^, ^12^ ^]^ and ectopic hepatic lipids.^[^ ^13^ ^]^ In a randomized crossover‐design trial, we tested whether adding a modest amount of LA‐rich oil to the diet (8 g high LA safflower oil per day), without altering other fats or impacting body‐weight change, affected body composition and glycemic control in postmenopausal women with type 2 diabetes.^[^ ^14^, ^15^ ^]^ Trunk adipose tissue^[^ ^14^ ^]^ and C‐reactive protein^[^ ^15^ ^]^ were decreased while lean mass and insulin sensitivity^[^ ^14^ ^]^ were increased in the women. These data corroborate the associative evidence that LA is useful in preventing and treating cardiometabolic disease.

Dietary LA intake may be decreasing in regions of the world where high oleic acid (OA) vegetable oils are replacing traditional LA‐rich oils in foods. In fact, the intake of both essential fatty acids, LA and alpha‐linolenic acid (18:3n‐3), may be inadequate for adults and children^[^ ^16^, ^17^ ^]^ as high OA oils grow in usage.^[^ ^17^, ^18^, ^19^ ^]^ Previous findings from our lab^[^ ^15^ ^]^ and others^[^ ^20^ ^]^ have revealed that increasing plasma or serum LA by ≈10% using LA‐oil fortification is sufficient for improvements of lipidemia, glycemia, insulin sensitivity, inflammation markers, trunk adipose mass, visceral adipose, and hepatic lipid accumulation in adults with type 2 diabetes and/or central obesity.

Dysregulated mitochondrial function is a central etiological factor contributing to many aspects of cardiometabolic diseases. Cardiolipin (CL), an inner mitochondrial membrane (IMM) phospholipid,^[^ ^21^ ^]^ plays an important role in mitochondrial respiration.^[^ ^22^, ^23^, ^24^, ^25^ ^]^ Tetralinoleoyl‐CL (LA_4_CL) is the LA‐rich species that predicts for efficient mitochondrial respiration in skeletal and cardiac muscle^[^ ^26^, ^27^, ^28^, ^29^ ^]^ and liver.^[^ ^30^ ^]^ Loss of LA_4_CL from the IMM reduces cellular respiration.^[^ ^29^, ^31^ ^]^ Many mitochondrial disorders result in reduced LA_4_CL in skeletal^[^ ^26^, ^27^, ^32^, ^33^ ^]^ and heart muscles.^[^ ^27^, ^32^ ^]^ In rodents, LA_4_CL is reduced in the livers of obese mice^[^ ^34^ ^]^ and the hearts of mice with diabetes.^[^ ^35^ ^]^ Dietary fortification with LA‐rich oils increased the LA content of CL in heart^[^ ^36^, ^37^, ^38^, ^39^, ^40^ ^]^ and liver^[^ ^37^, ^38^ ^]^ in rodent models. Adults with obesity, diabetes, or age‐associated muscle atrophy have lower LA_4_CL in skeletal muscle compared to “healthy” peers.^[^ ^41^, ^42^, ^43^ ^]^ Improving insulin sensitivity using exercise increases LA_4_CL.^[^ ^41^, ^43^ ^]^ To our knowledge, whether dietary fortification with LA‐oil increases LA_4_CL levels in adults has not been reported. This study aimed to compare the effect of dietary fortification with LA‐oil (7.4 g LA per day in 10 g oil per day) on 1) LA and OA in plasma, erythrocytes, and peripheral blood mononuclear cells (PMBC); and 2) LA_4_CL in PBMC. As an exploratory measurement, the effect of LA‐oil on cytokines associated with inflammation and metabolism was assessed.

## 2 Results

### 2.1 Participant Characteristics

**Figure 1:**
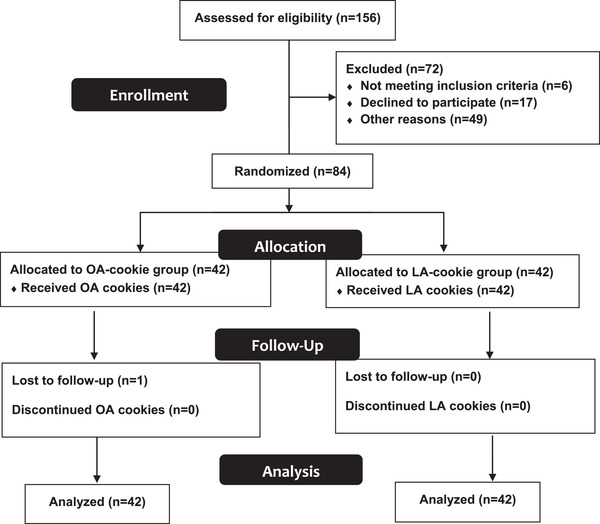
Flowchart of participants through the trial. LA, linoleic acid; OA, oleic acid.

Eighty‐four adults were randomized to either the OA‐cookie (*n* = 42) or LA‐cookie groups (*n* = 42) (**Figure** **1**). Participant characteristics are presented in **Table** **1**. In both the OA‐cookie and LA‐cookie groups, almost two thirds of the participants were female and the average age was 32 and 33 years, respectively. Accrual was terminated after reaching primary goal of 26 per group as funding was also limited. This differs from the goal at the onset of the trial where we had planned to accrue as noted in trial registry of *N* = 123. There were no differences in BMI (*p* = 0.94), glucose (*p* = 0.39), insulin (*p* = 0.30), and HOMA‐IR (*p* = 0.32) between the OA‐cookie and LA‐cookie groups. For both groups, the average BMI was in the overweight category and the average fasting glucose was under 100 mg dL^−1^.

**Table 1:** Participant characteristics

	OA‐Cookie (*N* = 41)	LA‐Cookie (*N* = 42)
Male^a)^	15 (35.7)	13 (31.7)
Female^a)^	27 (64.3)	28 (68.3)
Age^b)^	32.4 ± 14.2	32.8 ± 13.9
BMI^b)^	25.8 ± 5.0	25.7 ± 5.4
Glucose (mg dL^−1^)^b)^	95.8 ± 8.9	97.5 ± 9.7
Insulin (mIU mL^−1^)^b)^	9.7 ± 4.9	11.0 ± 6.6
HOMA‐IR^b)^	2.3 ± 1.3	2.7 ± 1.8
Education^a)^		
High school diploma/GED	3 (7.1)	4 (9.8)
Some college	9 (21.4)	8 (19.5)
2‐year or 4‐year college degree	18 (42.9)	8 (19.5)
Some graduate school	3 (7.1)	6 (14.6)
Master's degree or Doctoral Degree	9 (21.4)	15 (36.6)
Race^a)^		
Black	4 (9.5)	1 (2.4)
White	34 (81.0)	37 (90.2)
Asian	3 (7.1)	3 (7.3)
Other	1 (2.4)	0 (0.0)
Ethnicity^a)^		
Hispanic/Latino	5 (11.9)	1 (2.4)
Not Hispanic/Latino	37 (88.1)	40 (97.6)

^a)^ Data presented are *N* (%).

^b)^ Data are mean ± standard deviation.

*n* = 41–42 in the OA‐cookie group and the LA‐cookie group. Significant differences between groups for BMI, glucose, insulin, and HOMA‐IR (*p* < 0.05) are represented by asterisk (*) using student's *t*‐test.

BMI, body mass index; GED, General Educational Development; HOMA‐IR, Homeostatic model assessment of insulin resistance.

### 2.2 Cookie Consumption

The average self‐reported adherence to consuming one cookie per day was >96% (Table S3, Supporting Information) and was not different between groups (*p* = 0.13). Close to two thirds of the participants in both groups reported adding the cookies without altering their diets. About twice the number of participants in the LA‐cookie group reported substituting the cookie in place of another food than in the OA‐cookie group (LA = 19.4% vs OA = 8.3% participants). 16.7% and 25% of participants in the LA‐cookie and OA‐cookie groups respectively reported both adding the cookies and substituting the cookies in place of another food during the 2‐week intervention.

### 2.3 Fatty Acids

Fatty acids were analyzed at week 0 and week 2 in plasma, erythrocyte, and PBMC (**Table** **2**). At week 0, LA and OA content of each blood fraction were well‐balanced between the groups. Two weeks of LA‐cookie consumption increased LA in plasma, erythrocytes, and PBMC while 2 weeks of OA‐cookie consumption did not influence LA in the blood. The largest percent increase of LA was in PBMC, showing a 10% increase after 2 weeks of LA‐cookie consumption, followed by about a 7% increase in erythrocyte LA and 6% increase in plasma LA.

**Table 2:** Fortification with LA‐rich or OA‐rich oil foods alters OA and LA composition of plasma, erythrocytes, and PBMCs

	Week 0	Week 2	Δ (Week 2–Week 0)	*p*‐value	*p*‐value interaction	Adjusted *p*‐value interaction
Plasma oleic acid (18:1n‐9)						
OA‐Cookie	17.6 ± 2.6	18.0 ± 2.8	0.4 ± 2.2	0.18	<0.01	0.01
LA‐Cookie	17.7 ± 2.9	16.7 ± 2.4	‐1.1 ± 1.8	<0.01		
Plasma linoleic acid (18:2n‐6)						
OA‐Cookie	32.7 ± 3.9	32.6 ± 4.1	0.1 ± 3.0	0.93	<0.01	0.02
LA‐Cookie	32.9 ± 4.1	35.0 ± 3.8	2.1 ± 3.0	<0.001		
Erythrocyte oleic acid (18:1n‐9)						
OA‐Cookie	12.8 ± 1.0	13.3 ± 1.1	0.5 ± 0.6	<0.001	<0.001	<0.006
LA‐Cookie	13.1 ± 1.3	12.9 ± 1.1	‐0.2 ± 0.6	0.07		
Erythrocyte linoleic acid (18:2n‐6)						
OA‐Cookie	13.8 ± 1.5	13.8 ± 1.3	0.1 ± 1.2	0.71	<0.01	0.01
LA‐Cookie	13.6 ± 1.5	14.5 ± 1.8	0.9 ± 1.2	<0.001		
PBMC oleic acid (18:1n‐9)						
OA‐Cookie	10.1 ± 2.2	10.9 ± 1.8	0.7 ± 2.0	0.01	<0.01	0.02
LA‐Cookie	10.6 ± 1.7	10.2 ± 1.3	‐0.4 ± 1.6	0.15		
PBMC linoleic acid (18:2n‐6)						
OA‐Cookie	6.1 ± 1.4	6.0 ± 1.1	‐0.1 ± 1.5	0.59	0.02	0.11
LA‐Cookie	6.0 ± 1.1	6.6 ± 1.3	0.6 ± 1.3	<0.01		

Data presented are mean ± standard deviation. In the OA‐cookie group *n* = 42 at week 0 and *n* = 39 at week 2. In the LA‐cookie group *n* = 41–42 at week 0 and *n* = 41 at week 2. Significant differences between groups is established at *p* < 0.05 using linear mixed model for changes over time in each group and group × week interaction. Adjusted *p*‐values for the group × week interaction were determined using Bonferroni correction for multiple comparisons. One participant from the LA‐cookie group was identified as an outlier (greater than three standard deviations above the mean) at week 0 for PBMC LA and subsequently removed from PBMC fatty acid analysis. When the outlier was included, there was no difference in the overall change in PBMC LA between the cookie groups (*p* = 0.24) and no change in PBMC LA levels in the LA‐cookie group (*p* = 0.21); there was still significant difference in the change of PBMC OA between the cookie groups where there was a significant increase in PMBC OA in the OA‐cookie group (*p* = 0.01).

LA, Linoleic acid; OA, Oleic acid; PBMC, peripheral blood mononuclear cells.

In the LA‐cookie group, OA levels decreased in plasma but not erythrocytes or PBMC. In the OA‐cookie group, erythrocyte and PBMC OA increased but plasma OA did not increase. When the group by week *p*‐values were adjusted for multiple comparisons results were similar except there was no difference in the change of PMBC LA between the LA‐cookie and OA‐cookie group (*p* = 0.11).

### 2.4 Cardiolipin Species

**Figure 2:**
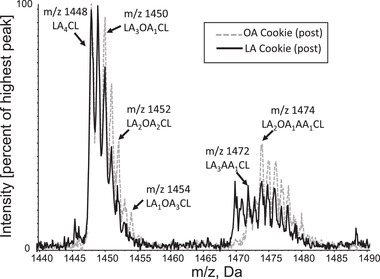
Representative mass spectrometry spectra of cardiolipin from post OA cookie PBMC (dotted gray line) and LA cookie PBMC (solid black line) with measured peaks and major species of those peaks are identified. AA, arachidonic acid; CL, cardiolipin; Da, daltons; LA, linoleic acid; *m*/*z*, mass to charge ratio; OA, oleic acid.

The major CL species of PBMC are depicted in **Figure** **2**. At week 0, CL species were well balanced between groups (**Table** **3**). After 2 weeks of consuming one cookie per day, LA_4_CL increased by about 5% in the PBMC of LA‐cookie group while LA_4_CL in the OA‐cookie group remained unchanged. There was a significant group by week interaction for LA_3_OA_1_ CL between the LA‐cookie and OA‐cookie groups (*p* < 0.01) with a significant decrease in the LA‐cookie group (*p* = 0.02). Although the LA‐cookie decreased LA_2_OA_2_ CL, the change in this CL species was not significantly different between the groups over the 2 weeks (*p* = 0.58). There were no differences in LA_1_OA_3_ CL between the two groups (*p* = 0.29). When the group by week *p*‐values were adjusted for multiple comparisons results were the similar. There were no significant group by week interactions for LA_3_AA_1_ CL or LA_2_OA_1_AA_1_ CL (data not shown).

**Table 3:** LA‐rich oil alters cardiolipin species in PBMCs

	Week 0	Week 2	Δ (Week 2–Week 0)	*p*‐value	*p*‐value interaction	Adjusted *p*‐value interaction
(18:2)_4_ CL, *m*/*z* 1448						
OA‐Cookie	59.9 ± 6.0	58.5 ± 7.0	‐0.8 ± 5.3	0.31	<0.01	0.01
LA‐Cookie	60.3 ± 6.4	63.5 ± 5.8	3.1 ± 5.7	<0.01		
(18:2)_3_(18:1)_1_ CL, m/z 1450						
OA‐Cookie	27.2 ± 4.1	28.8 ± 5.5	1.5 ± 4.8	0.09	<0.01	0.02
LA‐Cookie	27.6 ± 4.9	25.6 ± 4.6	‐1.9 ± 4.8	0.02		
(18:2)_2_(18:1)_2_ CL, *m*/*z* 1452						
OA‐Cookie	10.7 ± 2.5	10.1 ± 3.0	‐0.9 ± 3.2	0.22	0.58	1.0
LA‐Cookie	10.0 ± 2.9	8.9 ± 2.1	‐1.2 ± 3.2	0.03		
(18:2)_1_(18:1)_3_ CL, *m*/*z* 1454						
OA‐Cookie	2.1 ± 1.9	2.6 ± 1.5	0.3 ± 1.9	0.23	0.29	1.0
LA‐Cookie	2.1 ± 1.8	2.0 ± 1.3	0.0 ± 1.6	0.80		

Data presented are mean percent ± standard deviation. Percent was calculated from the *m*/*z* 1448, 1450, 1452, and 1454. In the OA‐cookie group *n* = 33 at week 0 and *n* = 30 at week 2. In the LA‐cookie group *n* = 37 at week 0 and *n* = 35 at week 2. Differences between groups were considered significant if *p* < 0.05 using linear mixed model for changes over time in each group and group × week interaction. Adjusted *p*‐values for the group × week interaction were determined using Bonferroni correction for multiple comparisons. 18:2 or LA, Linoleic acid; 18:1 or OA, Oleic acid; CL, cardiolipin; PBMC, peripheral blood mononuclear cells.

Some studies have demonstrated that BMI and gender may alter blood levels of polyunsaturated fatty acids.^[^ ^44^, ^45^ ^]^ When BMI or gender were tested for affecting the cumulative levels of LA in PBMCs, erythrocytes or plasma, and LA_4_CL, neither variables modified the effect of diet group on changes of LA or LA_4_ CL.

### 2.5 Biochemical Measurements

There were no significant differences at week 0 between the groups for total or HMW adiponectin, TNFr‐2, or LBP in this relatively healthy and nonobese adult cohort. There were also no significant groups by week interactions for any of the measurements (**Table** **4**).

**Table 4:** Changes of cytokines in OA and LA diet groups

	Week 0	Week 2	Δ (Week 2–Week 0)	*p*‐value	*p*‐value interaction	Adjusted *p*‐value interaction
Total adiponectin						
OA‐Cookie	6.3 ± 2.7	6.2 ± 2.7	0.0 ± 1.3	0.97	0.67	1.0
LA‐Cookie	5.7 ± 2.6	5.9 ± 3.1	0.1 ± 1.9	0.57		
HMW adiponectin						
OA‐Cookie	4.0 ± 2.5	4.1 ± 2.4	0.1 ± 1.1	0.72	0.84	1.0
LA‐Cookie	3.8 ± 2.3	3.8 ± 2.7	0.0 ± 1.9	0.93		
TNFr‐2						
OA‐Cookie	4694 ± 1807	4462 ± 1696	‐127 ± 1150	0.43	0.26	1.0
LA‐Cookie	4674 ± 1484	4196 ± 1227	‐485 ± 1328	0.02		
LBP						
OA‐Cookie	2720 ± 1412	2751 ± 1246	‐85 ± 1362	0.88	0.46	1.0
LA‐Cookie	3212 ± 1322	2957 ± 1100	‐249 ± 1069	0.23		

Data presented are mean ± standard deviation. In the OA‐cookie group *n* = 36–42 at week 0 and *n* = 33–39 at week 2. In the LA‐cookie group *n* = 40–42 at week 0 and *n* = 33–41 at week 2. Differences between groups were considered significant if *p* < 0.05 using linear mixed model for changes over time in each group and group × week interaction. Adjusted *p*‐values for the group × week interaction were determined using Bonferroni correction for multiple comparisons. HMW, high molecular weight; LA, Linoleic acid; LBP, lipopolysaccharide‐binding protein; OA, oleic acid; TNFr‐2, tumor necrosis factor receptor 2.

## 3 Discussion

In the present study, dietary LA (vs dietary OA) fortification for 2 weeks increased LA in plasma, erythrocyte and PBMC and LA_4_ CL in PBMC. Most studies testing the effects of LA fortification last from 2 to 16 weeks in duration lead to an increase in serum, plasma, or erythrocyte LA.^[^ ^15^, ^46^, ^47^, ^48^, ^49^ ^]^ We believe this to be the first study to analyze the change of cardiolipin species in PBMCs in response to daily fortification with less than one serving of oil (10 g oil per day).

The magnitude of change of LA in PBMC was greater than that of change of LA in plasma or erythrocytes. The PBMC fraction is composed of a mixture of lymphocytes, monocytes, and macrophages with turnover in the blood between 3 and 10 days. Because the PBMC blood cell fraction may serve as a useful surrogate marker for conditions related to cardiometabolic disease,^[^ ^50^, ^51^ ^]^ circadian misalignment,^[^ ^52^ ^]^ and biological aging,^[^ ^53^ ^]^ the change of PBMC LA may serve as an additional useful biomarker for future clinical investigations in dietary fatty acids, mitochondrial respiration, and human health.

To our knowledge, this is the first clinical trial to test the effect of a LA‐rich oil to alter the mitochondrial lipidome and specifically the cardiolipin phospholipid species. Because of the reliance of mitochondrial respiration on cardiolipin species in the IMM, these findings could have a profound impact on mitochondrial‐related disorders and diseases. In a previous study, cultured human fibroblasts grown in media supplemented with LA showed an increase of LA_4_CL.^[^ ^54^ ^]^ It will be important to understand whether there are metabolic implications of the increased amount of LA_4_ CL in human PBMC since increasing LA_4_ CL in animal tissues results in increased mitochondrial respiration.^[40,67]^. In addition, whether LA‐rich oil (10 g per day) supplementation increases LA_4_ CL in mitochondria‐rich tissues such as muscle, liver, adipose, and cardiac muscle in humans will be an important future study.

Dietary LA impacts mitochondrial respiration in muscles in preclinical rodent models of mitochondria dysfunction.^[^ ^40^, ^55^ ^]^ In a recent study, dietary LA partially restored LA_4_CL and muscle contractile force phenotype in skeletal muscle of mice deficient of tafazzin.^[67]^ In rats with heart failure, the reduction of LA_4_CL and mitochondrial respiration was prevented when fed a diet with LA.^[^ ^40^ ^]^ In humans, exercise, and weight loss both increase LA_4_CL in muscle tissues. In men and women who lost weight through moderate intensity exercise, LA_4_CL increased and LA_2_OA_2_ CL decreased in the vastus lateralis muscle; the changes of cardiolipin species were accompanied by an increase in mitochondrial respiration and improved insulin sensitivity.^[^ ^43^ ^]^ Our observation that supplementing the diet with a LA‐rich oil to increase blood levels of LA and LA_4_CL could explain how LA supplementation increased lean muscle mass, reduced trunk adipose, and improved glycemic control in postmenopausal women with type 2 diabetes.^[^ ^14^ ^]^ Evaluating whether LA fortification increases lean mass and improves insulin sensitivity coincident with increasing LA_4_CL and increasing mitochondrial respiration will be needed to understand the significance of a dietary LA‐oil changing PBMC cardiolipin species.

In the OA‐cookie group, OA levels increased in erythrocytes and PBMCs but not in plasma. OA is the main MUFA in the U.S. diet, representing 1/3 of all fats (or ≈12% total energy intake) consumed by adults.^[^ ^56^ ^]^ Replacing SFA with MUFA is associated with a reduced risk of CVD in some studies,^[^ ^3^, ^4^ ^]^ but not CHD.^[^ ^57^ ^]^ Previously, we have reported that erythrocyte OA was negatively correlated with markers of inflammation but had no relationship with adipose mass or other measures of body composition or insulin sensitivity.^[^ ^58^ ^]^ Increasing OA consumption through dietary oils may reduce total cholesterol and LDL‐cholesterol in men and women.^[^ ^59^, ^60^, ^61^ ^]^ However, to our knowledge, neither plasma OA levels^[^ ^62^ ^]^ nor MUFA intake^[^ ^63^ ^]^ have been associated with reducing diabetes risk.

In the present study, CL species were not changed in the OA‐cookie group. OA‐rich olive oil has been shown to increase OA in cardiolipin in the liver^[^ ^64^ ^]^ and cardiac muscle^[^ ^65^, ^66^ ^]^ in rats. While studies in humans examining the effect of OA intake on CL species are lacking, weight loss resulting from caloric restriction increased the percent of LA_3_OA_1_ CL but had no effect or slightly decreased LA_4_ CL in skeletal muscle of men and women.^[^ ^43^ ^]^ In contrast, exercise training increased LA_4_CL significantly without changing LA_3_OA_1_ CL and significantly increased mitochondrial respiration and fatty acid oxidation.^[^ ^43^ ^]^ Whether higher levels of OA fortification increase OA‐rich cardiolipin species and the impact of OA‐enriched CL, e.g., LA_3_OA_1_ CL, LA_2_OA_2_ CL, and LA_1_OA_3_ CL species, on energy metabolism in humans is not known.

The lack of effect of LA or OA on changes in adiponectin, TNFr‐2, or LBP in this study was not surprising given the short duration of this study and/or relatively healthy participant population. Many clinical interventions evaluating the effect fatty acid supplementation on plasma markers of metabolism and inflammation last between 4 and 24 weeks.^[^ ^15^, ^46^, ^47^, ^48^, ^49^ ^]^ In addition, adipokines and cytokines are usually altered in people with central obesity, type 2 diabetes, and/or the metabolic syndrome.^[^ ^67^, ^68^, ^69^, ^70^ ^]^ Our previous studies revealed that LA supplementation increased total adiponectin within 4 weeks in postmenopausal women with MetS^[^ ^71^ ^]^ and by 12 weeks in postmenopausal women with type 2 diabetes.^[^ ^15^ ^]^ Additionally, others have shown that TNFr‐2 was lowered after 10 weeks of a high LA diet compared to a high SFA diet in abdominally obese adults.^[^ ^20^ ^]^ Future studies comparing LA and OA oils in populations might need to be longer in duration and/or might have a more profound effect in people with central obesity, dysregulated metabolism, and/or elevated markers of inflammation.

The increase in blood LA after 2 weeks combined with the >95% self‐reported adherence to daily cookie consumption, demonstrates that using the cookies was a feasible method for delivering dietary oils to fortify habitual diets with a goal of increasing LA status in the blood. Other foods that have been used to deliver dietary oils rich in LA include muffins,^[^ ^72^, ^73^ ^]^ cake, frozen meals,^[^ ^74^ ^]^ and beverages.^[^ ^61^, ^75^ ^]^

There are several limitations to this study. First, as was already discussed, this study was relatively short in duration. Many interventions testing the cardiometabolic effects of dietary oils span many weeks to several months.^[^ ^15^, ^20^, ^71^, ^76^ ^]^ The short duration may explain the exceptionally high adherence and low drop‐out rates. A longer study is needed to determine if consuming a similar dose of LA for longer than 2 weeks is feasible and if a longer duration additively increases PBMC LA_4_ CL and PBMC levels of LA. Second, the participants in this study were generally healthy and therefore the results reported here cannot be generalized to other adult populations that have obesity, central obesity, insulin resistance, cardiometabolic disease risks, and mitochondrial conditions in tissues such as muscle and liver. Third, dietary assessment was not conducted in this study so the total amount of LA consumed in the diet before and during the daily cookie consumption is not known. Fourth, since no specific instruction was given to the participants on how to incorporate the daily cookie into their habitual diets, it is possible that other nutrients were altered during the 2‐week intervention. Finally, no measurement of masking or blinding was used in this study; therefore, it is possible participants were not masked to the intervention type (LA‐cookie vs OA‐cookie).

In conclusion, to our knowledge, this is the first study to demonstrate that dietary supplementation with less than one serving of LA‐rich oil per day increases LA in PBMC cardiolipin, e.g., LA_4_CL, as well as LA levels. These findings merit further examination into the effects on mitochondrial respiration in PBMC and potential impact on energy metabolism. Patients with obesity, cardiometabolic disease, and other conditions related to mitochondrial dysfunction may be a future cohort that should be studied.

## 4 Experimental Section

### Study Design and Participants

The study was a randomized double‐masked placebo‐controlled study that occurred between April 2016 and August 2017, approved by the Ohio State University Institutional Review Board (2015H0209) and registered with clinicaltrials.gov (NCT02841618; The Healthy Cookie Study: https://clinicaltrials.gov/ct2/show/NCT02841618). CONSORT reporting guidelines were used as applicable.^[^ ^77^ ^]^ A partial description of the study design, participant inclusion and exclusion criteria, and methods was described previously.^[^ ^44^ ^]^ Written informed consent was provided by all participants. Healthy adults (≥18 years old) were recruited from the Columbus, Ohio area. Participants were excluded from enrollment for tobacco use, food allergies/intolerances, current or previous diagnosis of diabetes, heart, kidney, liver or circulatory diseases, current treatment for cancer, gastric bypass surgery, gastrointestinal diseases, and pregnancy or nursing. A research product coordinator, who did not interact with participants, used a block randomization scheme generated to achieve 1:1 assignment for each of the two groups (Excel, Microsoft, Redmond, WA, USA). The research product coordinator also prepared packaged cookies for each subject and maintained the participant group code until unblinding.

In preparation for each study visit, participants were asked to fast for at least 10 h. After arriving to the Nutrition Research clinical room at the Ohio State University campus, participants were settled in a phlebotomy chair for the venous blood collection. At the first study visit (week 0) after the blood draw, participants were randomly assigned to either the LA cookie group (LA‐cookie) or the OA cookie group (OA‐cookie), which served as the comparative control group. The clinical research coordinator, other research care personnel and participants were masked to the treatments until the completion of the fatty acid analysis for plasma, erythrocyte, and PBMC by gas chromatography. The clinical research coordinator instructed participants to consume one cookie per day during the 2‐week intervention. Participants were asked to track their cookie consumption each day through 1) self‐reports using a daily check‐off form and 2) their return of uneaten cookies, if any, at the final study visit (week 2). The cookie check‐off form provided data for date, time of day, cookie identification number, and if the entire cookie was consumed. No specific instructions were given about how to incorporate the cookies into habitual diets; therefore, participants were asked about how the cookies were incorporated into their diets at the 2‐week study visit. Fasting blood samples were collected at each visit. Body mass index (BMI) was calculated from height and weight measured at week 0 using a wall‐mounted stadiometer and digital scale, respectively.

### Preparation of Blood Fractions

Plasma and erythrocyte samples were prepared from ETDA tubes (Becton, Dickinson and Company, Franklin Lakes, NJ, USA) that were stored on ice immediately after collection and until centrifugation at 1932 × *g* for 10 min at 4 °C. PBMC samples were prepared from CPT tubes (Becton, Dickinson and Company, Franklin Lakes, NJ, USA) according to the manufacturer's instructions.

### Cookies

The cookies were prepared in batches using a standardized recipe (Table S1, Supporting Information) with 10 g of either grapeseed oil (LA‐cookie; Pompeian, Inc., Baltimore, MD, USA) or high oleic safflower oil (OA‐cookie; Ventura Foods LLC, Brea, CA, USA). The LA‐cookie provided ≈7.4 g of LA per cookie and the OA‐cookie provided ≈7.7 g of OA per cookie. The fatty acid composition of the two oils was presented in Table S2, Supporting Information. Five flavors using an extract (blueberry, chocolate, lemon, peanut, cinnamon) and a topping (dried blueberries, chocolate chips, lemon glaze, peanuts, white chocolate chips) were available for LA‐cookie and OA‐cookie cookies. The nutrient composition (Table S2, Supporting Information) averaged across flavors was determined using Nutrient Database Systems for Research (Version 2016, University of Minnesota, Minneapolis, MN, USA) and the fatty acid profile was determined by gas chromatography. All cookies contained a similar nutrient profile with the exception of the amount of monounsaturated and polyunsaturated fats.

At week 0, each participant was given a sample of the five cookie flavors and asked to choose the cookie flavors to consume during the 2‐week intervention. After the 2‐week intervention, the number of cookies each participant reported consuming, even if the entire cookie was not consumed, as well as the number of cookies each participant was expected to consume was used calculate adherence which was reported as the percent of expected cookie consumption.

### Dosage Information and Regimen

Participants were instructed to consume one cookie per day for 2 weeks. Each cookie contained 10 g of oil which was less than one serving of oil per day (15 g). The dose of oil was chosen based on prior studies showing an effect of safflower oil on lean mass, adiponectin, and glycemic control in women with type 2 diabetes.^[^ ^14^, ^15^ ^]^

### Fatty Acid Composition Analyses of Cookies and Blood Fractions

Random samples of the OA‐cookie and LA‐cookie cookies without the topping were selected for fatty acid analysis to verify consistency between batches (data not shown). In addition, plasma, erythrocytes, and PBMC samples were prepared from fasting blood samples at each visit for fatty acid composition. Fatty acid analysis was conducted via gas chromatography as previously described.^[^ ^44^, ^71^, ^78^ ^]^ In brief, total lipids from plasma, PBMC, and the cookies were extracted with 2:1 (v/v) chloroform:methanol^[^ ^79^ ^]^ and methylated using 5% hydrochloric acid in methanol.^[^ ^80^ ^]^ Fatty acids in erythrocyte were extracted and methylated using 14% boron trifluoride in methanol.^[^ ^81^, ^82^, ^83^ ^]^ Column oven conditions and flow rate of the carrier gas were as previously reported^[^ ^78^ ^]^ or when the oven temperature reached 220 °C it was lowered to 210 °C at a rate of 5 °C min^−1^. Retention times of each fatty acid was compared to purchased standards (Matreya, LLC, Pleasant Gap, PA, USA and Nu‐Check Prep Inc., Elysian, MN, USA) and fatty acids were reported as percent of total identified.^[^ ^71^, ^78^ ^]^

### Cardiolipin Species Analyses

Cardiolipin species were quantified in PBMCs with liquid chromatography coupled to electrospray ionization mass spectrometry in an API 4000 mass spectrometer (Sciex, Framingham, MA, USA) according to previously published methods.^[^ ^84^ ^]^ PBS (100 μL) was added to PBMC pellets and lipids extracted according to previously published methods with 1 mmol tetramyristal‐cardiolipin as an internal standard (Avanti Polar Lipids, Alabaster, AL, USA).^[^ ^84^, ^85^ ^]^ There were six major species of CL identified in the PBMC samples which were listed in Table S4, Supporting Information with the spectra shown in Figure 2. For the primary evaluation, only CL having OA and LA side chains were presented. These data were expressed as a percent of four species with a mass to charge ratio (*m*/*z*) of 1448 (LA_4_CL), 1450 (LA_3_OA_1_CL), 1452 (LA_2_OA_2_CL), and 1454 (LA_1_OA_3_CL) (Table S4, Supporting Information) with fatty acyl side chains confirmed with tandem mass spectrometry (data not shown). Two other arachidonic acid containing species having an *m*/*z* of 1472 and 1474 were expressed as a percent of all six species before analysis.

### Biochemical Analysis of Plasma

Fasting plasma levels of insulin and glucose were measured at week 0 while tumor necrosis factor‐receptor 2 (TNF‐r2), lipopolysaccharide‐binding protein (LBP), and total and high molecular weight (HMW) adiponectin were measured at week 0 and week 2. Insulin, glucose, TNFr‐2, and LBP were analyzed by the Clinical Research Core laboratories (Center for Clinical and Translational Science, The Ohio State Wexner Medical Center). Insulin, TNFr‐2, and LBP were measured with a MESO QuickPlex SQ 120 using electrochemilluminescence Meso Scale Discovery kits (Gaithersburg, MD, USA). Glucose was measured using the Dimension Xpand Clinical Chemistry System (Siemens Medical Diagnostics, Decatur, GA, USA), a modification of a previously described method.^[^ ^86^ ^]^ The homeostatic model assessment of insulin resistance (HOMA‐IR) was calculated from the fasting insulin and glucose values.^[^ ^87^ ^]^ Total and HMW adiponectin were measured using ELISA kits from Alpco (Salem, NH, USA).

### Statistical Analysis

A power calculation for the change of plasma LA was conducted. In our previous study we saw an increase of 2.8% ± 0.8% in plasma LA content after a 16‐wk long intervention delivering 8 g LA‐rich safflower oil per day by capsule.^[^ ^15^ ^]^ We then conducted In a two‐week, single treatment arm pilot study to detect the magnitude of change of erythrocyte content of LA in response to daily consumption of a healthy cookie delivering 10 g LA‐rich grapeseed oil per day (R.M. Cole, dissertation). At the baseline visit, average erythrocyte LA = 14.1% ± 1.7% and final erythrocyte LA = 14.5% ± 1.6% in N = 41 healthy adults; therefore, N = 42 adults per treatment group (multiple of 4 for block randomization) was selected for the present study.

The primary goal of this study was to determine whether a modest addition of an oil rich in LA could change of LA content in plasma, erythrocytes, and PBMCs. The secondary goal was to determine if the LA‐rich oil could alter cardiolipin species in PBMCs. Exploratory measurements were to assess the effect of the LA‐rich oil on cytokines. A student's *t*‐test was used to assess differences in BMI and markers of glycemia between the two cookie groups at baseline. A two‐sample Wilcoxon rank‐sum test was used to assess differences in adherence between the two cookie groups. To assess the change in the study measurements in each group during the intervention and compare them between groups, linear mixed models with random intercepts were fit to each measure accounting for the correlation over time within each participant. Linear regression was used to determine if gender or BMI influenced the change in LA in each blood fraction and in PBMC LA_4_CL. Significance was established at *p* < 0.05. For linear mixed Models *p*‐values were reported as unadjusted and adjusted for multiple comparisons using Bonferroni correction. Statistical analyses were conducted with Stata Version 15 (StataCorp LLC, College Station, TX, USA).

## Conflict of Interest

M.A.B. has a research award from the United States Soybean Board for a project unrelated to this study; R.M.C. has partial support from the United States Soybean Board. M.A.B. is a scientific consultant for the Bath and Body Works for concepts unrelated to this study. Other authors have no conflicts of interest to declare.

## Author Contributions

M.A.B. and R.M.C. conceived the study, designed the research trial and wrote the drafts and final versions of the manuscript. R.M.C. formulated the study foods, was blinded to study treatments and led the day‐to‐day study intervention and data coordination. A.A. assisted with study food preparation and oversaw product packaging and distribution and was unblinded to the study treatments. R.M.C. conducted biochemistry analysis of blood samples. G.S. conducted the mitochondrial lipidome analyses for cardiolipins. A.N. oversaw all statistical model design and data analyses. All authors discussed the findings, interpretations, and cowrote the final versions of the paper.

## Supporting information

Supporting Information

## Data Availability

Data described in the manuscript, code book, and analytic code will be made available upon request pending application and approval.
