# Supplementary material for: Linoleic Acid‐Rich Oil Alters Circulating Cardiolipin Species and Fatty Acid Composition in Adults: A Randomized Controlled Trial
Source: Mol Nutr Food Res. 2022 Jun 21;66(15):2101132. doi: 10.1002/mnfr.202101132 (PMC9540417; doi:10.1002/mnfr.202101132)
Supplement: Supplementary file 1 — Supporting Information [file MNFR-66-2101132-s001.pdf]

Linoleic Acid-rich Oil Alters Circulating Cardiolipin Species and Fatty Acid Composition in Adults: A Randomized Controlled Trial

Rachel M Cole, et al.,

Supplementary Tables

Supplementary Table 1. Formula, Nutrient and Fatty Acid Composition of Cookies

Supplementary Table 2. Fatty Acid Composition[1] of the Dietary Oils Used in the Cookies

Supplementary Table 3. Adherence and Pattern of Consumption of Cookies

Supplementary Table 4. Major cardiolipin species identified in peripheral blood mononuclear cells.

Supplementary Table 1. Formula and Nutrient Composition of Cookies

Nutrient per cookie	OA-Cookie	LA-Cookie
Energy (kcal)	229 ± 1.6	229 ± 1.6
Total fat (g)	12 ± 0.2	12 ± 0.2
Saturated fat (g)	1 ± 0.1	1 ± 0.1
Polyunsaturated fat (g)	2 ± 0.0	8 ± 0.2
Monounsaturated fat (g)	8 ± 0.1	2 ± 0.1
Total carbohydrates (g)	28 ± 0.2	28 ± 0.2
Dietary fiber (g)	2 ± 0.1	2 ± 0.1
Sugars (g)	12 ± 0.2	12 ± 0.2
Protein (g)	4 ± 0.1	4 ± 0.1
**Fatty Acid Composition of cookies (%)**		
Palmitic acid (16:0)	7.0 ± 0.3	7.9 ± 0.7
Stearic acid (18:0)	1.9 ± 0.1	3.4 ± 0.2
Oleic acid (18:1n9)	71.8 ± 1.6	17.9 ± 1.0
Linoleic acid (18:2n6)	17.4 ± 1.6	69.0 ± 1.6
Other	1.9 ± 0.1	1.8 ± 0.1
**Fat Distribution (%)**		
Saturated fatty acids	9.4 ± 0.4	11.5 ± 0.8
Monounsaturated fatty acids	72.8 ± 1.6	18.9 ± 1.0
Polyunsaturated fatty acids	17.8 ± 1.6	69.6 ± 1.5

Nutrients were determined using NDSR 2016. kcal: Kilocalorie; LA: Linoleic acid; NDSR, Nutrient Database Systems for Research. OA: Oleic acid. Fatty acid analysis was performed by gas chromatography on samples of the cookies without the topping. Other fatty acids, each

compromising <1% of the total fatty acids, were 14:0, 16:1n7, 18:1n7, 18:3n3, 20:0, 20:1n9, 20:4n6. LA: Linoleic acid; OA: Oleic acid. Data presented are mean ± standard deviation.

Supplementary Table 2. Fatty Acid Composition[1] of the Dietary Oils Used in the Cookies.

	OA-Safflower Oil			Grapeseed Oil		
Palmitic acid (16:0)	5.0	±	0.2	6.2	±	0.2
Stearic acid (18:0)	1.8	±	0.1	3.3	±	0.1
Oleic acid (18:1n9)	77.4	±	0.6	15.5	±	0.6
Linoleic acid (18:2n6)	14.1	±	0.5	73.6	±	0.6
Other	1.7	±	0.1	1.5	±	0.0

[1]Analyses were performed by gas chromatography. Data presented are the mean percent ± standard deviation. Other fatty acids, each comprising <1% of the total fatty acids, were 14:0, 16:1n7, 18:1n7, 18:3n3, 20:0, 20:1n9, 22:0. OA: Oleic acid.

Supplementary Table 3. Adherence and Pattern of Consumption of Cookies

	OA-Cookie	LA-Cookie
Adherence	96.6 ± 8.2	99.0 ± 2.6
Added the cookies without altering diet, % (n)	24 (66.7)	23 (63.9)
Substituted the cookies in place of another food, % (n)	3 (8.3)	7 (19.4)
Both (added and substituted), % (n)	9 (25.0)	6 (16.7)

Self-reported adherence data are presented as mean percent of expected cookie consumption ± standard deviation; n=36 in the OA-cookie group and n=40 in the LA-cookie group. All other data presented as n (%); n=36 per group. No significant difference in adherence was found between groups using a two-sample Wilcoxon rank-sum test. LA: Linoleic acid; OA: Oleic acid.

Supplementary Table 4. Major cardiolipin species in PBMC

m/z	Dominant fatty acid acyl composition	Abbreviation
1448	$(18:2)_4$ CL	LA_4 CL
1450	$(18:2)_3$ $(18:1)_1$ CL	LA_3OA_1 CL
1452	$(18:2)_2$ $(18:1)_2$ CL	LA_2OA_2 CL
1454	$(18:2)_1$ $(18:1)_3$ CL	LA_1OA_3 CL
1472	$(18:2)_3$ $(20:4)_1$ CL	LA_3AA_1 CL
1474	$(18:2)_2$ $(18:1)_1$ $(20:4)_1$ CL	$LA_2OA_1AA_1$ CL

18:2 or LA, Linoleic acid; 18:1 or OA, Oleic acid; 20:4 or AA, Arachidonic acid. CL: cardiolipin.
